# Genetic evaluation of sheep for resistance to gastrointestinal nematodes and body size including genomic information

**DOI:** 10.5713/ajas.19.0816

**Published:** 2020-04-12

**Authors:** Tatiana Saraiva Torres, Luciano Silva Sena, Gleyson Vieira dos Santos, Luiz Antonio Silva Figueiredo Filho, Bruna Lima Barbosa, Antônio de Sousa, Fábio Barros Britto, José Lindenberg Rocha Sarmento

**Affiliations:** 1 Department of Animal Science, Federal University of Piauí, Teresina, PI 64049-550, Brazil; 2 University Unity of Campos Belos, State University of Goiás (UEG), Campos Belos, GO 73840-000, Brazil; 3 Department of Animal Science, Federal University of Piauí, Bom Jesus, PI 64900-000, Brazil; 4 Federal Institute of Education, Science and Technology of Maranhão, Caxias, MA 65609-899, Brazil; 5 Technical College of Teresina, Federal University of Piauí, Teresina, PI 64049-550, Brazil; 6 Department of Biology, Federal University of Piauí, Teresina, PI 64049-550, Brazil

**Keywords:** Accuracy, Genetic Parameters, Rump Height, ssGBLUP, Worm Infection

## Abstract

**Objective:**

The genetic evaluation of Santa Inês sheep was performed for resistance to gastrointestinal nematode infection (RGNI) and body size using different relationship matrices to assess the efficiency of including genomic information in the analyses.

**Methods:**

There were 1,637 animals in the pedigree and 500, 980, and 980 records of RGNI, thoracic depth (TD), and rump height (RH), respectively. The genomic data consisted of 42,748 SNPs and 388 samples genotyped with the OvineSNP50 BeadChip. The (co)variance components were estimated in single- and multi-trait analyses using the numerator relationship matrix (**A**) and the hybrid matrix **H**, which blends **A** with the genomic relationship matrix (**G**). The BLUP and single-step genomic BLUP methods were used. The accuracies of estimated breeding values and Spearman rank correlation were also used to assess the feasibility of incorporating genomic information in the analyses.

**Results:**

The heritability estimates ranged from 0.11±0.07, for TD (in single-trait analysis using the **A** matrix), to 0.38±0.08, for RH (using the **H** matrix in multi-trait analysis). The estimates of genetic correlation ranged from −0.65±0.31 to 0.59±0.19, using **A**, and from −0.42±0.30 to 0.57±0.16 using **H**. The gains in accuracy of estimated breeding values ranged from 2.22% to 75.00% with the inclusion of genomic information in the analyses.

**Conclusion:**

The inclusion of genomic information will benefit the direct selection for the traits in this study, especially RGNI and TD. More information is necessary to improve the understanding on the genetic relationship between resistance to nematode infection and body size in Santa Inês sheep. The genetic evaluation for the evaluated traits was more efficient when genomic information was included in the analyses.

## INTRODUCTION

Gastrointestinal nematode infections (GNI) are one of the main constraints to sheep production, due to the high costs of anthelmintic treatments, as well as the body growth delay and mortality of severely affected animals [1,2].

The strategies for control of GNI rely mainly on treatments with anthelmintic drugs, which are generally expensive and labor intensive. In addition, these drugs may leave residues in meat and milk, and are subject to the resistance of worms to their active ingredients [2]. Due to the spread of drug resistant endoparasites, researchers and producers have been using the selection of resistant animals as one of the main alternatives to minimize the effect of GNI on production systems [3].

Studies on resistance to gastrointestinal nematodes are usually based on traits that in dicate worm infection, for example, color of the ocular conjunctiva, number of eggs per gram of feces, and body condition score (BCS). An alternative to quantify the effect of these characteristics together consists in using them to compose the resistance to GNI (RGNI) trait, using computational intelligence based on Fuzzy logic [4].

In order to better assess the genetic potential of resistant individuals, another important aspect to be considered is the evaluation of the relationship between parasitic resistance and production traits. For instance, to assess the impact of parasitic infections on body development of farmed animals under natural rearing conditions, an option is to estimate (co)variance components and genetic correlations between traits that indicate body development and traits that are indicative of the level of parasitic infection [5]. Thus, the investigation on the relationship between traits such as chest depth, rump height (RH), and RGNI in sheep may help in the selection for resistance to parasite infections based on body size, that is easier to measure.

Accurate estimates of genetic parameters for the traits of interest are essential for selection of genetically superior animals. In this sense, a multiple-trait model is expected to increase the accuracy of genetic parameters by making use of information from genetically correlated traits [6]. Furthermore, the inclusion of genomic data in the models will contribute to better estimation of variance components, genetic parameters and breeding values, thus favoring the selection of superior animals based on more accurate information [7].

Studies on RGNI and body size in sheep using genomic information are still scarce. To the best of our knowledge, there are no reports on the relationship between RGNI and body size in Santa Inês sheep. Therefore, the objective of this study was to estimate (co)variance components, genetic parameters and (genomic) breeding values for gastrointestinal nematode resistance and body size in Santa Inês sheep using the numerator relationship matrix (**A**) and its combination with the genomic relationship matrix (**G**), i.e., the **H** matrix, to evaluate the efficiency of including genomic information in the genetic evaluation for the traits in study.

## MATERIALS AND METHODS

### Animal care

All the experimental procedures involving animals were approved by the Committee on Ethics in the Use of Animals (CEUA) of the Federal University of Piauí, Brazil (protocol number 414/2017).

### Population description

Pedigree data from 1,637 Santa Inês sheep were available. A total of 206 individuals were sires and 713 were dams. The pedigree depth included eight generations, where 933 animals had both their parents known, 40 individuals had only dam information, and 664 had unknown ancestry. Data were collected from 2012 to 2018, in 18 farms located in the Mid-North sub-region of Brazil (states of Piauí and Maranhão). The animals were registered with the Brazilian Association of Sheep Breeders (ARCO) or belonged to the sheep and goat conservation nucleus of the federal research unit Embrapa Meio-Norte (Campo Maior, Piauí, Brazil). The farms used semi-intensive system, in which the animals were raised receiving feed supplementation during the dry season. This system differed slightly across farms and time period, but this was accounted for in the statistical models.

### Phenotypic data

Fecal samples were taken directly from the rectum of the animals, using plastic bags which were then stored into Styrofoam boxes containing ice gel packs until the samples were taken to the laboratory. The parasitological evaluation was performed within 24 hours after the feces collection, through the fecal egg count (FEC) adopting the procedures described by Ueno and Gonçalves [8].

The assessment of the color degree of the conjunctival mucous membrane was performed using the FAMACHA method [9]. The BCS was assessed by palpation of the prominences of the spinous and transverse bones of the spine, fat coverage, and muscle development between the last rib and the ileum wing, according to the procedures described by Russel et al [10].

The combination of FAMACHA score, BCS, and FEC values was used to generate a grade ranging from 0 to 10 (continuous scale) that represented the RGNI trait for each animal. This grade was calculated using the CAPRIOVI software [11], based on Fuzzy logic. FEC values were used on the raw scale (non-transformed) of egg counts per gram of feces to obtain the value of RGNI. Only information from animals with records of FAMACHA score, BCS, and FEC in a collection performed on the same date were used to generate RGNI values. It was assumed that higher RGNI represented higher animal’s resistance to gastrointestinal nematode infection.

Two body dimensions were used to assess the relationship of body size with RGNI. The following measurements were obtained using a tape measure graduated in centimeters, while the animals were standing on a flat surface in an appropriate position: RH, measured as the distance from the highest point over the sacrum to the ground; and thoracic depth (TD), calculated as the difference between withers height and the distance from the sternum (between the fore legs) to the ground.

### Genomic data

The blood samples used for DNA extraction were collected by puncture of the jugular vein using a needle attached to vacuum tubes containing ethylenediaminetetraacetic acid anticoagulant. The DNA extraction was performed using the Qiagen DNeasy Blood &Tissue kit according to the manufacturer’s protocol. The DNA quality was checked using 1% agarose gels with 1× sodium borate buffer during 60 minutes at 60 V. The digitalization and visualization of the gels were performed using an L-PIX photo documenter (Loccus Biotecnologia, Cotia, SP, Brazil). The NanoDrop 3300 Fluorospectrometer was used for the quantification of DNA, using the AccuBlue Broad Range dsDNA Quantitation Kit (Biotium Inc., Fremont, CA, USA). Prior to genotyping, the extracted DNA was kept frozen (−20°C).

A total of 389 samples were genotyped using the Ovine SNP50 BeadChip (Illumina Inc., San Diego, CA, USA), which contains 54,241 single nucleotide polymorphisms (SNPs).

### Editing and quality control of phenotypic and genomic data

Phenotypic records from animals with no information of birth year, farm, sex, and age were removed from further analyses. The contemporary groups for all traits were defined by the effects of year (2012, 2013, 2014, 2017, and 2018) and season of data collection (January to May: rainy season; June to December: dry season), sex, and farm. The age of the animal at the day of measurement was fitted as a covariate (linear effect). After data editing, only contemporary groups with at least two animals were kept. A total of 500, 980, and 980 records of RGNI, TD, and RH, respectively, were used for the analyses. Data of animals aged at least six months were considered.

The genotyping quality control was performed using the PREGSF90 program [12]. Quality control consisted of excluding SNPs with unknown genomic positions (n = 682), located on sex chromosomes (n = 1,472) or mitochondrial DNA (n = 3), with minor allele frequency lower than 0.05 (n = 5,009), call rate lower than 95% (n = 4,319), and in extreme departure from Hardy-Weinberg equilibrium (p<10^−6^, n = 8). In addition, samples with call rate lower than 0.90 were removed. After the quality control, 42,748 SNPs and 388 samples remained for further analyses.

### Statistical analyses

The (co)variance components were estimated via Bayesian inference in single- and multi-trait analyses, using the animal model, by the pedigree-based BLUP and single-step genomic BLUP (ssGBLUP) procedures. The GIBBS1F90 program [12] was used for these analyses. The posterior distributions of the (co)variance components were obtained using the POSTGIBBSF90 program [12]. The analyses consisted of a chain of 1,000,000 iterations with a burn-in of 200,000 cycles, and thinning interval of 100 iterations. Thus, 8,000 samples were used to obtain the (co)variance estimates and posterior standard deviation.

The estimates of variance components were used as starting values for calculation of (genomic) estimated breeding values, i.e., (G)EBVs, using the BLUPF90 program [12].

The general model can be written as follows:

y=Xβ+Zα+e

in which: **y** is the vector of phenotypic records for the trait(s); β is the vector of fixed effects (contemporary group and age as a covariate); α is the vector of genetic additive direct effects of the animal, where $\alpha \sim \text{N}\;(0,A\sigma_{a}^{2})$, when only pedigree information is used, in single-trait models, and $\alpha \sim \text{N}\;(0,H\sigma_{a}^{2})$, using both pedigree and genomic information, where $\sigma_{a}^{2}$ is the genetic additive variance, **A** is the additive-genetic numerator relationship matrix, and **H** is the additive-genetic relationship matrix based on both pedigree and genomic information; **e** is the vector of residual effects, where $e\sim \text{N}\;(0,I\sigma_{e}^{2})$, in single-trait models; **X** is the incidence matrix of fixed effects; and **Z** is the incidence matrix of additive genetic effects.

For multi-trait analyses, the (co)variance structure among traits was modeled as follows:

$$\text{Var}\;\left[\begin{array}{c} \alpha \\ e \end{array}\right]=\left[\begin{array}{cc} A⊗G_{0} & 0\\ 0 & I⊗R_{0} \end{array}\right]$$

where

$$G_{0}=\left[\begin{array}{ccc} \sigma_{\alpha 1}^{2} & \sigma_{\alpha 1\alpha 2} & \sigma_{\alpha 1\alpha 3}\\ \sigma_{\alpha 2\alpha 1} & \sigma_{\alpha 2}^{2} & \sigma_{\alpha 2\alpha 3}\\ \sigma_{\alpha 3\alpha 1} & \sigma_{\alpha 3\alpha 2} & \sigma_{\alpha 3}^{2} \end{array}\right]$$

is the (co)variance matrix between traits due to animal additive genetic effects,

$$R_{0}=\left[\begin{array}{ccc} \sigma_{e1}^{2} & \sigma_{e1e2} & \sigma_{e1e3}\\ \sigma_{e2e1} & \sigma_{e2}^{2} & \sigma_{e2e3}\\ \sigma_{e3e1} & \sigma_{e3e2} & \sigma_{e3}^{2} \end{array}\right]$$

is the (co)variance matrix between traits due to random error effects, **I** is the identity matrix (N×N, where N is the number of observations for each trait), and ⊗ is the Kronecker product operator.

For the inclusion of genomic information in the models using the ssGBLUP procedure, **A**^−1^ was replaced by **H**^−1^ [13], calculated as follows:

$$H^{-1}=A^{-1}+\left[\begin{array}{cc} 0 & 0\\ 0 & G^{-1}-A_{22}^{-1} \end{array}\right]$$

where **A**^−1^ is the inverse of the numerator relationship matrix, **G**^−1^ is the inverse of the genomic relationship matrix, and $A_{22}^{-1}$ is the inverse of the pedigree-based relationship matrix for genotyped animals. When calculating **G**, we fixed α and β to 0.95 and 0.05, respectively, which are the default values of the PREGSF90 program to create a weighted **G** as 0.95**G**_0_+ 0.05**A**_22_ (to avoid singularity problems), in which **G**_0_ was created using the first method described by VanRaden [14], as follows:

$$G_{0}=\frac{(M-P)\;(M-{P)}^{\prime }}{2\sum_{\text{j}=1}^{\text{m}}{\text{p}}_{\text{j}}(1-{\text{p}}_{\text{j}})}$$

where: **M** is the allele-sharing matrix with *m* columns (*m* = total number of markers) and *n* lines (*n* = total number of genotyped animals); and **P** is a matrix containing the frequency of the second allele (p_j_), expressed as 2_pj_. The observed allele frequencies were used.

The theoretical accuracies of breeding values obtained in single- and multi-trait models using both relationship matrices (**A** and **H**) were calculated using the standard errors of prediction (SEP), estimated from the inverse of the mixed model equations as follows:

$${\text{Acc}}_{ij}=1-\sqrt{\left({\text{PEV}}_{ij}/\sigma_{a_{j}}^{2}\right)}$$

where PEV*_ij_* (prediction error variance) is equivalent to ${\text{SEP}}_{ij}^{2}$, in which SEP*_ij_* is the standard error of prediction calculated by the BLUPF90 program for the (G)EBV of animal *i* for the trait *j* and $\sigma_{a_{j}}^{2}$ is the additive genetic variance of the trait *j*.

The Spearman coefficients between the rank positions of each animal were calculated to investigate whether changes in the ranking of animals occurred based on their EBVs across the different scenarios using BLUP and ssGBLUP, in single-trait analyses.

## RESULTS AND DISCUSSION

The descriptive statistics for the traits in study are shown in Table 1. The highest coefficient of variation was observed for RGNI, as this characteristic is more subject to environmental variations in relation to body size traits.

The estimates of variance components differed according to the models used in both scenarios (with and without inclusion of genomic information). Only the estimates of additive genetic variance for RGNI decreased in multi-trait models in both scenarios (Table 2). This result is probably due to the smaller sample size for this characteristic, in comparison to the other evaluated traits.

Additive genetic variances were higher when genomic in formation was included in single- and multi-trait analyses for all traits. Estimates of residual variance decreased in all the scenarios in which genomic information was used.

Compared to the **A** matrix, the **G** describes the genetic similarity between individuals more accurately, which results in more accurate estimation of variance components [7]. In the evaluation of traits with few records, it is expected an increase in detection of genetic variance when genomic information is used, due to the correction of pedigree errors before building the relationship matrices [15]. Thus, it is expected that environmental variance estimates decrease for all traits by including genomic information in the single- and multi-trait models. This indicates better model fit and ability to genetically differentiate the best animals, as well as better estimation of genetic parameters.

In this study, heritability estimates (h ^2^) were classified as low (<0.20), moderate (0.20 to 0.40), and high (>0.40). For RGNI, heritability estimates were low when using the **A** matrix (Table 2). These values suggest that direct selection for this trait as formulated in the current study would have little benefit if the estimation of variance components were based only on phenotype and pedigree data. On the other hand, estimates of moderate magnitude obtained in the analyses which included genomic information indicate that selection for RGNI is feasible. Thus, it is recommended the use of genomic information, if possible, to better estimate variance components for this trait. According to Guo et al [6], traits with low heritability are the most benefited by genomic selection. In general, heritability estimates for traits that indicate resistance to endoparasites in sheep have a broad range, as they rely on nematode species, animal’s breed, environment, and methodological factors [16].

For TD, the heritability estimates obtained in the current study were low when using the **A** matrix in both scenarios (single- and multi-trait analyses). On the other hand, when using genomic information, the heritability estimates for TD had moderate magnitude in both scenarios (Table 2). Thus, selection for this trait will also be favored by the inclusion of genomic information.

The heritability estimates for RH in the current study were moderate in all scenarios (Table 2). These results suggest that the response to direct selection for this trait will yield satisfactory gains regardless of the inclusion of genomic information. In sheep, direct heritability estimates for body conformation traits, such as height, usually range from moderate to high [17,18]. Thus, the genetic improvement of these traits can be efficiently achieved through the classical methods. Nevertheless, studies using data derived from molecular markers can generate information that may be incorporated into genetic evaluations and selection indexes in the future [18]. Furthermore, the inclusion of genomic information allows the detection of higher proportion of additive genetic variance, as mentioned above, and consequently provides more accurate estimates of breeding value, even for traits with moderate or high heritability.

The advantage of using genomic data in the current study was evident, especially for traits with low heritability. Thus, direct selection for increased resistance to worm infection based on the trait RGNI proposed in this study will be more efficient when performed with the inclusion of genomic data.

Estimates of genetic correlation between the evaluated traits ranged from low to moderate magnitude (Table 3). For interpretation purposes, the genetic correlation estimates were categorized as low (<0.60), moderate (0.60 to 0.80), and high (>0.80). Estimates of genetic correlation between RGNI and body size traits were antagonistic and moderate when the **A** matrix was used. When the **H** matrix was used, the genetic correlation between RGNI and RH was antagonistic and low. There were changes in magnitude and direction of the genetic correlation between RGNI and TD obtained using genomic information, in comparison to the result obtained using the **A** matrix.

The antagonistic values of the genetic correlation estimates between RGNI and RH obtained in both scenarios suggest that taller Santa Inês sheep tend to show reduced RGNI. In addition, the correlation between RGNI and TD using the **H** matrix (0.04±0.35) suggests that genes involved in thoracic development have almost no effect on RGNI. Nevertheless, there were relatively few records of the RGNI trait in the current study, even in relation to the other evaluated traits. Thus, more studies using higher number of RGNI records are necessary for more accurate assessment of the genetic relationship between RGNI and the body size traits evaluated in this study, in Santa Inês sheep.

Estimates of genetic correlation between parasitic resistance indicator traits, such as FEC or FAMACHA score, and production traits are favorable if negative [5]. As in the present study it was assumed that higher values of RGNI indicate greater resistance to worm infection, only the genetic correlation between RGNI and TD using genomic information was favorable, although close to zero.

In an experimental herd of Santa Inês sheep (n = 119) submitted to two natural challenges, Lôbo et al [19] obtained near-zero genetic correlations between *Haemonchus contortus* egg counts per gram of feces and body weight from weaning to 550 days of age. These authors suggested that selection for resistance should not have an unfavorable effect on the growth potential of sheep. In a study with Santa Inês sheep from different herds, Oliveira et al [20] obtained favorable genetic correlation estimates between body weight (n = 2,487) and FAMACHA score (−0.40±0.17, n = 2,489), egg counts (logFEC+1) of *H. contortus* (−0.27±0.17, n = 1,569), and body condition score (0.84±0.08, n = 2,516).

In the present study, unfavorable genetic correlation estimates between RGNI and the body size traits indicate that there may be negative influence of selection for RGNI on body size of Santa Inês sheep, or decrease in parasitic resistance due to selection of bigger animals. This is evidenced by the estimates of genetic correlation between RGNI and RH in both scenarios (including or not genomic information). However, as mentioned above, to better understand the genetic relationship between body size and the RGNI trait in Santa Inês sheep, further information on these phenotypes is needed. In general, there are still divergences between different studies regarding the relationship of parasitic resistance and characteristics that indicate body development in sheep.

According to Stear et al [5], a justificative for unfavorable genetic correlations between traits that indicate resistance to worms and production traits is that, under certain circumstances, the immune response to gastrointestinal nematodes may have deleterious effects on body growth in sheep. Coltman et al [21] claimed that the sign of the genetic correlation may also depend on environmental conditions, so that the abundance of resources favorable to animal nutrition and sanitary control of herds decreases the incidence of infections, and this makes difficult the generalization and extrapolation of results between different studies.

Most of the studies with sheep have shown favorable genetic correlation estimates between resistance to gastrointestinal parasites and production traits [22,23]. However, there are few results on the relationship between body size and parasitic resistance [24]. In the literature, only Coltman et al [21] reported estimates of genetic correlation between body size and parasitic resistance in small ruminants. These authors obtained favorable genetic correlations between the rear leg length and FEC (logarithmic scale) ranging from −0.31±0.13 to −0.22±0.04, in *Teladorsagia circumcincta* infected wild Soay sheep, in Scotland. According to these authors, the genetic relationship between body development and parasitic resistance in the Soay sheep breed is natural as it is a wild breed. Therefore, the results of Coltman et al [21] require caution in their extrapolation to domestic breeds, especially those raised in tropical climate conditions, where nematodes such as *H. contortus* are predominant.

Domestic sheep breeds underwent different selection processes over time and, according to Coltman et al [21], these processes resulted in factors that led to inconsistent genetic correlations between parasitic resistance and productive traits reported in different studies, with different breeds or with the same breed in different environments. According to Greer [25], inconsistent results of the relationship between parasitic resistance and productivity in sheep may be due to differences in the relative selection pressures applied for productive traits, allele frequencies in each population, the composition of parasitic challenge or the environment in which the measures were collected, that may result in variations in defense mechanisms that differ in their cost and effectiveness in controlling parasites.

On the other hand, the relationship between body size traits in sheep is consistent between different studies. In the present study, estimates of genetic correlation between TD and RH without inclusion of genomic information (0.59±0.19) or including genomic data in the analysis (0.57±0.16) were favorable. These positive values suggest that there are genes in common that act on the expression of these two traits, so that the selection for one will result in an increase in the other due to the pleiotropy effect. However, the low magnitude of these estimates suggests that selection for increase in TD based on RH (and vice versa), in Santa Inês sheep, may have little efficiency.

In other sheep breeds, the estimates of genetic correlation between body height and chest dimensions range from low to high. Janssens and Vandepitte [17], for example, reported estimates of genetic correlation of 0.67, 0.59, and 0.53 between body depth and withers height in Bleu du Maine, Suffolk, and Texel sheep, respectively. In this same study, the authors found genetic correlations between body height and chest circumference ranging from 0.39 to 0.68. Estimates of genetic correlation between RH and chest circumference of 0.46 and 0.38±0.22 were obtained, respectively, by Jafari and Hashemi [26], in Makuie sheep, and by Hossein-Zadeh and Ghahremani [27], in Moghani sheep.

Estimates of theoretical accuracy of breeding value, in the present study, ranged from 0.046 for RGNI, using a single-trait model with the BLUP method, to 0.188 for RH, using a multi-trait model with the ssGBLUP method (Table 4). Gains in accuracy were observed for all the evaluated traits when genomic information was used in the models. These gains are due to the correct estimation of genetic relationship between animals and the increase in variance partitioning when genomic information is added to the models [28].

In general, the highest accuracy estimates were observed when the multi-trait models with the inclusion of genomic information were used (Table 4). An increase in the accuracy of (G)EBVs is expected with the use of multi-trait models due to the availability of information of genetically related traits [6]. This allows the use of more information to evaluate the genetic potential of animals and, consequently, results in more accurate breeding values for the evaluated traits, due to the reduction of the prediction error variances [29]. According to these authors, the multi-trait model will result in more benefits for traits that have low heritability and small number of observations.

However, in the current study, the greatest gains in accuracy when comparing the results obtained using models that included genomic information to those obtained using the **A** matrix were achieved using single-trait models. The lowest gains in accuracy using a multi-trait genomic model were observed for RGNI, probably because this trait showed antagonistic or close to zero genetic correlations with the other traits. According to Pszczola et al [30], it is expected that the gain in accuracy of breeding value for a trait with few records is greater when it is strongly correlated with the predictor trait. In the case of the population evaluated in the current study, it would be interesting to increase the amount of RGNI records, as well as the number of genotyped animals. Thus, it would be expected to obtain higher and more reliable additive genetic (co)variance estimates, as well as more accurate estimates of (G)EBVs for RGNI.

**Figure 1:**
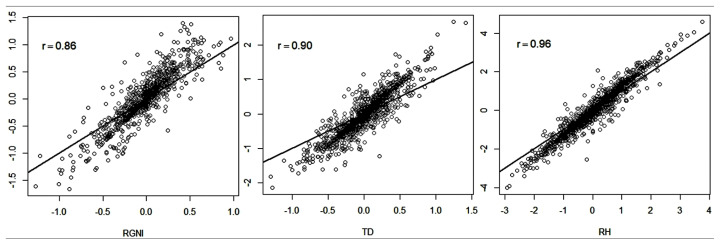
Estimated breeding values derived from BLUP (axis x) and single-step GBLUP (axis y), and Spearman ranking correlation (r) for resistance to gastrointestinal nematode infection (RGNI), thoracic depth (TD), and rump height (RH).

Spearman correlations between the ranks of animals had high magnitude using the BLUP and ssGBLUP methods (Figure 1). These results indicate that the classification of animals according to their (G)EBVs diverged slightly when adopting the use of genomic information.

The largest dispersion of (G)EBVs was observed for RGNI (Figure 1), which had the lowest rank correlation (0.86). For TD and RH, there were lower variations in the rank of the animals based on their (G)EBVs. Six of the animals used for the analyses had genotype information and had no phenotypic information of RH and TD, whereas a total of 59 genotyped animals had no information for RGNI. According to McManus et al [16], in situations where there are genotyped animals without progeny and phenotypic information, the ssGBLUP method increases the accuracy of genetic evaluations and there are changes in animal rankings compared to results obtained using the BLUP method. According to these authors, this is because the BLUP method relies only on family information for the calculation of EBVs, while the ssGBLUP method uses the genomic information (SNPs) of the animals that were genotyped but do not have phenotype information. Thus, because TD and RH had more phenotype and genotype information than RGNI, the change in classification of animals based on the predictions performed with the evaluated methods was smaller when compared to that observed in the RGNI trait.

In conclusion, the correlation estimates between animal ranks according to their EBVs obtained using the methods in study, as well as the superiority of accuracy estimates of breeding values observed using the ssGBLUP method reinforce the idea that the inclusion of genomic information for the genetic evaluation of these traits will be favorable and, therefore, it should be used, whenever possible, to obtain more reliable results.

The inclusion of genomic information will benefit the direct selection for the evaluated traits, especially RGNI and TD. The genetic evaluation of Santa Inês sheep for these traits will be benefited by using genomic data, due to the increase in accuracies. More information is necessary to improve the understanding on the genetic relationship between resistance to nematode infection and body size in Santa Inês sheep.

## Figures and Tables

**Table 1:** Descriptive statistics for resistance to gastrointestinal nematode infection, thoracic depth, and rump height

Trait	N	Mean	SD	CV	Minimum	Maximum
RGNI (grade)	500	5.65	2.15	37.98	1.94	8.05
TD (cm)	980	34.67	3.19	9.19	24.00	48.50
RH (cm)	980	75.56	3.79	5.01	61.00	92.00

SD, standard deviation; CV, coefficient of variation; RGNI, resistance to gastrointestinal nematode infection; TD, thoracic depth; RH, rump height.

**Table 2:** Estimates of variance components and heritability for resistance to gastrointestinal nematode infection and body size traits in Santa Inês sheep using different relationship matrices

Traits	Single-			Multi-		
	
	$\sigma_{\text{a}}^{2}$	$\sigma_{\text{e}}^{2}$	h^2^±PSD	$\sigma_{\text{a}}^{2}$	$\sigma_{\text{e}}^{2}$	h^2^±PSD
**A**						
RGNI	0.52	2.74	0.16±0.08	0.38	2.89	0.12±0.07
TD (cm)	0.84	6.85	0.11±0.07	1.14	6.66	0.15±0.06
RH (cm)	2.91	7.27	0.28±0.08	3.26	7.12	0.31±0.08
**H**						
RGNI	0.88	2.43	0.26±0.09	0.70	2.63	0.21±0.08
TD (cm)	1.59	6.20	0.20±0.08	1.73	6.17	0.22±0.08
RH (cm)	3.85	6.49	0.37±0.08	4.05	6.47	0.38±0.08

Single-, single-trait analysis; Multi-, multi-trait analysis; $\sigma_{\text{a}}^{2}$, additive genetic variance; $\sigma_{\text{e}}^{2}$, residual variance; h^2^, heritability estimate; PSD, posterior standard deviation; **A**, numerator relationship matrix; RGNI, resistance to gastrointestinal nematode infection; TD, thoracic depth; RH, rump height; **H**, hybrid matrix combining the genomic matrix with the numerator relationship matrix.

**Table 3:** Genetic correlations between resistance to gastrointestinal nematode infection and body size traits in Santa Inês sheep using different relationship matrices

Traits	TD (cm)±PSD	RH (cm)±PSD
**A**		
RGNI	−0.65±0.31	−0.64±0.22
TD (cm)	-	0.59±0.19
**H**		
RGNI	0.04±0.35	−0.42±0.30
TD (cm)	-	0.57±0.16

TD, thoracic depth; PSD, posterior standard deviation; RH, rump height; **A**, numerator relationship matrix; RGNI, resistance to gastrointestinal nematode infection; **H**, hybrid matrix combining the genomic matrix with the numerator relationship matrix.

**Table 4:** Estimates of average accuracy of breeding values for Santa Inês sheep evaluated for resistance to gastrointestinal nematode infection and body size traits using different relationship matrices

Traits	A		H		GAS (%)	GAM (%)
	
	Single-	Mult-	Single-	Mult-		
RGNI	0.046	0.090	0.073	0.092	58.69	2.22
TD	0.060	0.098	0.105	0.115	75.00	17.35
RH	0.138	0.161	0.174	0.188	26.09	16.77

**A**, numerator relationship matrix; **H**, hybrid matrix combining the genomic matrix with the numerator relationship matrix; Single-, single-trait analysis; Multi-, multi-trait analysis; GAS, gain in accuracy in single-trait analysis with inclusion of genomic information; GAM, gain in accuracy in multi-trait analysis with inclusion of genomic information; RGNI, resistance to gastrointestinal nematode infection; TD, thoracic depth; RH, rump height.
